# Clinical significance of stroke nurse in patients with acute ischemic stroke receiving intravenous thrombolysis

**DOI:** 10.1186/s12883-021-02375-6

**Published:** 2021-09-16

**Authors:** Zhao-Han Xu, Qi-Wen Deng, Qian Zhai, Qing Zhang, Zhen-Jie Wang, Wen-Xia Chen, Meng-Meng Gu, Teng Jiang, Jun-Shan Zhou, Ying-Dong Zhang

**Affiliations:** Department of Neurology, Nanjing First Hospital, Nanjing Medical University, 210006 Nanjing, China

**Keywords:** Stroke nurse, Door-to-needle time, Acute ischemic stroke, Intravenous thrombolysis

## Abstract

**Background:**

Reports have proven that shorter door-to-needle time (DTN time) indicates better outcomes in AIS patients received intravenous thrombolysis. Efforts have been made by hospitals and centers to minimize DTN time in many ways including introducing a stroke nurse. However, there are few studies to discuss the specific effect of stroke nurse on patients’ prognosis. This study aimed to compare consecutive AIS patients before and after the intervention to analyze the effect of stroke nurse on clinical outcome of AIS patients.

**Methods:**

In this retrospective study, we observed 1003 patients from November 2016 to December 2020 dividing in two groups, collected and analyzed AIS patients’ medical history, clinical assessment information, important timelines, 90 mRS score, etc. Comparative analysis and mediation analysis were also used in this study.

**Results:**

A total of 418 patients was included in this study, and 199 patients were enrolled in the stroke nurse group and 219 was in the preintervention group. Baseline characteristics of patients showed no significant difference except there seems more patients with previous ischemic stroke history in the group of stroke nurse. (*p* = 0.008). The median DTN time significantly decreased in the stroke nurse group (25 min versus 36 min, *p* < 0.001) and multivariate logistic regression analysis showed the 90-day mRS clinical outcome significantly improved in the stroke nurse group (*p* = 0.001). Mediation analysis indicated the reduction of DTN time plays a partial role on the 90 days mRS score and the stroke nurse has some direct effect on the improvement of clinical outcome (*p* = 0.006).

**Conclusions:**

The introduction of stroke nurse is beneficial to clinical outcome of AIS patients and can be use of reference in other hospitals or centers.

## Background

Over the last 30 years, stroke has become the leading cause of adult-disability and death in China, which causes huge burden to family and society [1–3]. The incidence and morality of stroke has been increasing year by year, and ischemic stroke represents the majority of total strokes [1, 4]. Intravenous thrombolysis (IVT) has been proven to be an effective way to help patients gain reperfusion and improve the clinical outcomes of ischemic stroke [5–7]. However, this treatment is highly time-sensitive. The number of patients eligible for thrombolysis is limited due to the 4.5 h therapeutic window, and the effectiveness of IVT varies with time [5]. Door-to-needle time (DTN time) is defined as the time from hospital arrival to thrombolysis administration. It is a rather objective measurement of hospital management. Guidelines for the Early Management of Patients with Acute Ischemic Stroke indicates a target DTN time of less than 60 min [5]. Studies have shown that shorter DTN time was associated with lower disability, mortality and better outcomes [8, 9].

Therefore, efforts have been made by hospitals to reduce the DTN time [10–12]. However, due to the limitations of material resources and manpower, the median DTN time varies in different hospitals. The most common reasons for the time delay were failure to identify the eligible patients timely, requirement to control hypertension aggressively, and in-hospital delays [9].

Our stroke center of Nanjing First Hospital, Nanjing Medical University, a national advanced stroke center affiliated with Stroke Prevention Project, National Health.

Commission, has been arranging a stroke nurse to accompany each patient suspected to suffer from a stroke to go through the whole diagnosis and assessment process since January 2019. We hypothesized that the joining of a stroke nurse would reduce the time used to response and cut the procrastination in hospital. This study includes 418 patients to investigate whether this modification of protocol helps shorten the DTN time and improves the prognosis of AIS patients.

## Methods

We conducted a single-center retrospective study in the stroke demonstration center of Nanjing first hospital. Our center introduced the participation of the 24 h on-call stroke nurse in January 1, 2019. The role of the stroke nurse was to escort patients suspected to suffer from stroke during the emergency diagnosis and treatment. Once the stroke nurse was alerted, she would take initial history, help patients get assessment and imaging examinations and help neurologist on duty to review the indications and contraindications for recombinant tissue plasminogen activator (rt-PA). During the IVT process, the stroke nurse would monitor the patient’s symptoms and vital signs, such as heart rate, respiration and blood pressure. The stroke nurse’s work would be finished when she handed the patient to the inpatient department. There, the patient would get next phase of treatment.

**Fig. 1:**
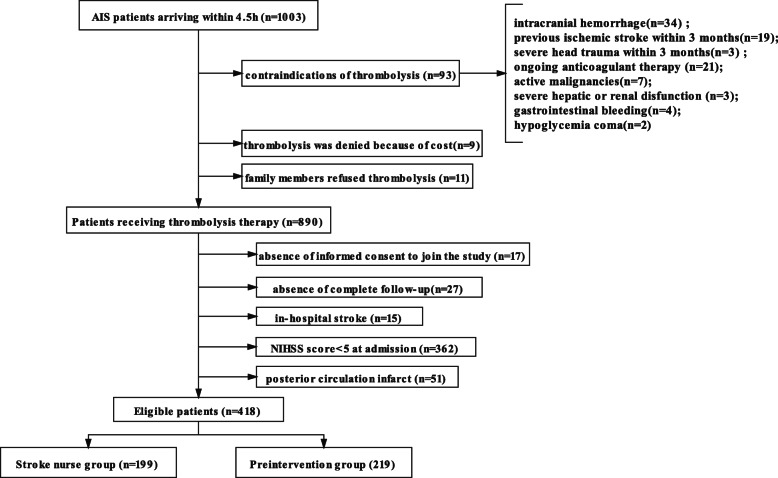
Flowchart of the study patients to illustrate study screening, recruitment, and follow-up

We observed 1003 consecutive acute ischemic stroke (AIS) patients who arrived within 4.5 h from November 2016 to December 2020 before and after the participation of the stroke nurse. The inclusion criteria were (1) AIS symptoms occurred within 4.5 h and received thrombolytic therapy [rt-PA 0.9 mg/kg] ;(2) NIHSS score ≥ 5 at admission;(3) age over 18 years old;(4) imaging evidence suggested anterior circulation infraction. The exclusion criteria were (1) absence of thrombolysis therapy including contraindications of thrombolysis (n = 93), thrombolysis was denied because of cost and family members refused thrombolysis; (2) absence of complete follow-up; (3) absence of informed consent to join the study; (4) in-hospital stroke; (5) NIHSS score < 5 at admission; (6) posterior circulation infarct. We excluded patients with NIHSS < 5 because we wanted to observe whether patients experienced early neurological improvement after treatment. Patients with posterior circulatory ischemia (POCI) was excluded because stroke severity was inaccurately assessed by the NIHSS in those patients and the proportion of those patients was small. Detailed information is shown in Fig. 1.

The baseline characteristics of patients were collected including: sex, age, significant medical history (previous ischemic stroke, hypertension, diabetes, and atrial fibrillation), cause of stroke (based on the Trial of ORG 10,172 in Acute Stroke Treatment (TOAST) classification), the glucose level at hospital arrival, the undergoing of endovascular therapy (EVT). We recorded the important duration, including time from stroke onset to hospital admission (onset-to-door time), from hospital admission to intravenous alteplase (DTN time) and from stroke onset to intravenous alteplase time (onset-to-needle time).

Each patient was assessed by professional neurologists using National Institute of Health Stroke Scale (NIHSS) score at the admission before intravenous (IV) thrombolysis administration, 1 h, 24 h, 72 h and 7 days from admission. A face to face/through phone follow-up assessment was arranged after 3 months using the modified Rankin Scale (mRS). We compared the important time consumption and patients’ conditions to find out if there were connections between the stroke nurse and patients’ prognosis.

### Statistical analysis

The data was analyzed by Statistical software SPSS 26.0 for Windows (SPSS Inc.,

Chicago, IL). Categorical variables such as medical history, sex and etiology were described by frequencies and percentages and variables of skewed distribution like the glucose level, durations, were expressed as medians (interquartile range [IQR]). The preintervention group was compared to the postintervention period by chi-square test for categorical variables, 2-tailed t-test for normally distributed variables and Mann-Whitney U-test for nonparametric and abnormally distributed variables. Variables with *P* < 0.05 of the univariable logistic regression analysis were also analyzed by multivariate logistic regression to obtain independent variables.

We also use the results of regression analysis and Bootstrap method to create mediation models to explore the significance of mediation effects among the join of stroke nurse, DTN time and 90 days mRS score.

### Ethics approval and consent to participate

This study policy was explained detailly and verbal informed consent was obtained from each study patient or their family members, which was approved by the ethics committee of Nanjing First Hospital, Nanjing Medical University and conducted in full accordance with the World Medical Association Declaration of Helsinki.

## Results

These 1003 patients with acute ischemic stroke are patients arriving within 4.5-hour thrombolysis time window. We excluded patients that didn’t receive thrombolysis because of thrombolysis contraindications (*n* = 93), denied because of cost (*n* = 9) and family members refused thrombolysis (*n* = 11) as shown in Fig. 1. Additionally, absence of informed consent to join the study (*n* = 17), absence of complete follow-up (*n* = 27), in-hospital stroke (*n* = 15), NIHSS < 5 (*n* = 362) and posterior circulation infarct (*n* = 51) were also excluded. Finally, a total of 199 consecutive AIS patients who underwent IV thrombolysis with the accompany of a stroke nurse (stroke nurse group) and 219 AIS patients without the stroke nurse (preintervention group) were included in this study. The baseline characteristics and demographics are shown in Table 1. There were no significant differences in the patients’ gender, age, treatment of embolectomy, initial mRS and NIHSS score. However, there seems more patients with previous ischemic stroke in the preintervention group (*p* = 0.008). The stroke nurse group shows longer onset-to-door time, the median DTN time significantly reduced from 36 min (interquartile range [IQR] 28–45) to 25 min ([IQR] 17–35) (*p* < 0.001). The two groups also share similar onset-to-needle time. Outcomes and logistic regression analyses are shown in Table 2 and Table 3. The results show the intervention of stroke nurse significantly improved the 90 days mRS score (*p* = 0.001). Early neurological improvement which was decided when NIHSS score reduced 4 scores after the IVT, showed no statistical difference between two groups.

**Table 1:** baseline characteristics and demographics

	All(418)	Stroke nurse group(199)	preintervention group(219)	*p*-value
Median age (IQR)-yr.	71(61–80)	70(61–80)	72(61–80)	0.518
Male sex- no. (%)	255(61.00)	124(62.31)	131(59.82)	0.602
Medical history-no. (%)				
Previous ischemic stroke	50(11.96)	15(7.54)	35(15.98)	0.008
History of atrial fibrillation	129(30.86)	53(26.63)	76(34.70)	0.074
History of diabetes mellitus	103(24,64)	46(23.12)	57(26.03)	0.490
History of hypertension	297(71.05)	140(70.35)	157(71.69)	0.763
mRS score of 0 ~ 2 before stroke onset-no. (%)	392(93.78)	191(95.98)	201(91.78)	0.076
Median glucose level at hospital arrival (IQR) -mmol/L	5.86(4.89–7.47)	6.01(4.96–7.51)	5.84(4.86–7.41)	0.660
Cause of stroke - no. (%)				0.380
Intracranial atherosclerosis	172(41.15)	88(44.22)	84(38.36)	
Cardioembolism	138(33.01)	67(33.67)	71(32.42)	
Small-vessel occlusion	78(18.66)	31(15.58)	47(21.46)	
Undetermined	30(7.18)	13(6.53)	17(7.76)	
Median duration (IQR)-min				
From stroke onset to hospital admission	100(60–155)	120(60–160)	90(55–150)	0.025
From hospital admission to intravenous alteplase	30(23–40)	25(17–35)	36(28–45)	< 0.001
From stroke onset to intravenous alteplase	135(95–191)	140(95–190)	135(95–192)	0.692
NIHSS score				
Median NIHSS score (IQR) before IVT	11(7–14)	11(7–14)	11(7–14)	0.999
Median NIHSS score (IQR) at 1 h after IVT	9(5–14)	8(5–14)	9(5–14)	0.270
Median NIHSS score (IQR) at 24 h after IVT	6(4–12)	6(3–11)	7(4–13)	0.198
Median NIHSS score (IQR) at 3d after IVT	5(2–11)	5(2–10)	5(2–11)	0.403
Median NIHSS score (IQR) at7d after IVT	4(1–9)	4(1–8)	3(1–9)	0.700
Treated with embolectomy no. (%)	145(34.69)	73(36.68)	72(32.88)	0.414

*IVT* intravenous thrombolysis; *NIHSS* National Institute of Health Stroke Scale

**Table 2:** Outcomes

	All(418)	Stroke nurse group (199)	preintervention group (219)	*p*-value
**Primary outcome**				
mRS at 90 days	2(0–4)	2(0–3)	2(1–4)	0.001
**Secondary outcomes**				
Clinical outcomes				
mRS score at 90 days according to range %				
0–1	175(41.87)	94(47.24)	81(36.99)	0.034
0–2	233(55.74)	121(60.80)	112(51.14)	0.047
0–3	307(73.44)	158(79.40)	149(68.04)	0.009
0–4	356(85.17)	184(92.46)	172(78.54)	< 0.001
0–5	375(89.71)	187(93.97)	188(85.84)	0.006
Death no. (%)	43(10.29)	12(6.03)	31(14.16)	0.006
Early neurological improvement no. (%)				
1 h after IVT	83(19.86)	45(22.61)	38(17.35)	0.178
24 h after IVT	166(39.71)	87(43.72)	79(36.07)	0.111
3d after IVT	228(54.55)	115(57.79)	113(51.60)	0.204
7d after IVT	288(68.90)	136(68.34)	152(69.41)	0.814

*mRS* modified Rankin Scale

**Table 3:** Logistic regression analyses

Outcome	Unadjusted effect (95 % CI)	*p*	Adjusted effect (95 % CI)	*p*
**Primary outcome**				
Median age (IQR)-yr	1.00(0.98–1.01)	0.553		
Male sex- no. (%)	1.11(0.75–1.65)	0.602		
mRS score of 0 ~ 2 before stroke onset-no. (%)	2.14(0.91–5.03)	0.082		
Median glucose level at hospital arrival (IQR) -mmol/L	0.98(0.91–1.06)	0.608		
Cause of stroke - no. (%)				
Intracranial atherosclerosis	1			
Cardioembolism	0.90(0.58–1.41)	0.648		
Small-vessel occlusion	0.63(0.37–1.08)	0.095		
Undetermined	0.73(0.33–1.59)	0.430		
History of ischemic stroke	0.43(0.23–0.81)	0.009		
History of atrial fibrillation	0.68(0.45–1.04)	0.075		
History of diabetes mellitus	0.85(0.55–1.34)	0.490		
History of hypertension	0.94(0.61–1.43)	0.763		
From stroke onset to hospital admission	1.00(1.00-1.01)	0.033		
From hospital admission to intravenous alteplase	0.97(0.96–0.98)	< 0.001		
From stroke onset to intravenous alteplase	1.00(1.00–1.00)	0.789		
Median NIHSS score (IQR) before IVT	1.01(0.97–1.04)	0.760		
Treated with embolectomy no. (%)	1.18(0.79–1.77)	0.414		
mRS at 90 days	0.84(0.76–0.93)	0.001	0.87(0.78–0.97)	0.016
**Secondary outcomes**				
Clinical outcomes				
mRS score at 90 days according to range				
0–1	1.53(1.03–2.25)	0.034	1.27(0.82–1.96)	0.280
0–2	0.67(0.46-1.00)	0.047	1.26(0.82–1.94)	0.294
0–3	1.81(1.16–2.83)	0.009	1.61(0.97–2.67)	0.067
0–4	3.35(1.81–6.21)	< 0.001	3.04(1.57–5.90)	0.001
0–5	2.57(1.28–5.16)	0.008	2.39(1.14–5.02)	0.021
Early neurological improvement				
1 h after IVT	1.39(0.86–2.25)	0.179	1.37(0.82–2.29)	0.227
24 h after IVT	1.38(0.93–2.04)	0.111	0.73(0.48–1.11)	0.146
3d after IVT	1.28(0.87–1.89)	0.205	1.32(0.88–1.98)	0.183
7d after IVT	0.95(0.63–1.44)	0.814	0.91(0.59–1.41)	0.680
Death	0.39(0.19–0.78)	0.008	0.42(0.20–0.88)	0.021

*IVT* intravenous thrombolysis; *NIHSS* National Institute of Health Stroke Scale, *mRS* modified Rankin Scale

**Fig. 2:**
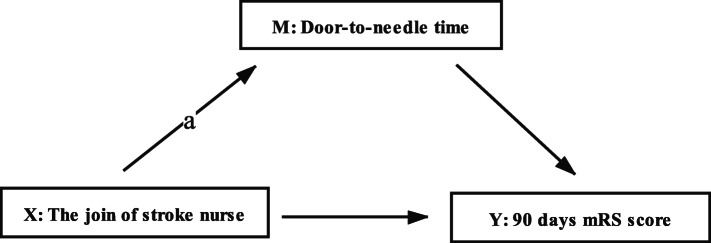
The created mediation models of the join of stroke nurse, door-to-needle-time and 90 days mRS score

The created mediation models and analysis results are shown in Fig. 2, Table 4 and Table 5. It turns out that DTN time plays a partial mediating effect on the 90 days mRS score, which indicates that the stroke nurse has direct influence on patients’ 90-day prognosis to some extent (*p* = 0.006).

**Table 4:** Mediating effect model analysis

		Standardized Coefficients	SE	t	95 % Confidence Interval for B		*p*
					LLCI	ULCI	
M	Constant a	51.26	3.04	16.88	45.29	57.23	
	X	-11.01	1.95	-5.65	-14.83	-7.18	
Y	Constant a	2.75	0.39	7.10	1.99	3.51	0.001
	M	-0.55	0.20	-2.76	-0.94	-0.16	
	X	0.01	0.00	1.77	0.00	0.02

**Table 5:** Mediating effect analysis

	Effect	Boot SE	t	*p*	95 % Confidence Interval	
					LLCI	ULCI
Total effect	-0.64	0.19	-3.34	0.001	-0.26	-0.32
Direct effect	-0.55	0.20	-2.76	0.006	-0.94	-0.16
Indirect effect(s)	-0.09	0.06			-0.22	0.00

## Discussion

Our single-center retrospective study showed that the introduction of stroke nurse improves clinical outcome of ischemic stroke patients regardless the partial mediating effect of reducing DTN time.

There is a general consensus that the earlier AIS patients get treatment, the better prognosis would be. There are three vital periods of time to evaluate: the onset-to-door time, DTN time and onset-to-needle time. The DTN time is considered to be measurable in the hospital management system. The latest guidelines for the early management for AIS patients released by the American Heart Association/American Stroke Association in 2018 suggested a primary goal of achieving DTN time within 60 min. However, many researches propose that shorter DTN time tends to indicate better outcomes and efforts have been made to reduce accelerate the access to thrombolysis [13]. There were studies conducted by centers to analyze DTN time by introducing a stroke nurse, response unit or designed code [11, 14–16]. Previous studies showed that good intervention could limit delays and improve DTN time [11, 16–19]. In our study, we found that from 2016 to 2020 the median DTN time significantly decreased from 36 to 25 min. The join of the stroke nurse helps to triage patients, communicate between patients and doctors, collect initial history, accelerate the physical and imaging examination, monitor and manage patients’ vital signs during the thrombolysis process. We believe these methods contributed to the improvement of DTN time too. Also, the stroke nurse group shows significant improvement in mRS score (p = 0.001), which indicates that the joining of stroke nurse may have an effect on AIS patients’ long-term prognosis.

We found that the preintervention group shows shorter onset-to-door time which results in the non-differential onset-to-needle time. Mediation models and analysis were performed to clarify whether the improvement of 90 days prognosis is mediated by DTN time. It turns out that the initiation of stroke nurse does have a positive effect on patients’ prognosis despite DTN time plays a partial mediating effect in the process. Thus, the implement of specialized and talented stroke nurse may have some positive effect on AIS patients and can be use of reference for other centers or hospitals.

The NIHSS is prevalently used to measure the primary clinical outcome for ischemic stroke patients [20, 21] and mRS is used in the long-term functional assessment [22, 23]. In this study, we accessed patients’ neurological conditions at admission, 1 h after the treatment, day 1, day3 and day7. We define the reduction of NIHSS score ≥ 4 as improvement in neurological functions within 7 days to see if the stroke nurse helps to improve patents’ early functional recovery. There were no statistical differences between groups.

There are several limitations in our study. This is a retrospective study, although a quite large sample size of patients was analyzed, there was a possibility of bias due to the selection, treatment and assessment of patients. Second, we only focused on ischemic patients with obstruction in the anterior circulation, which account for the majority of the location of intracranial artery occlusion, may also lead to deviation.

## Conclusion

The introduction of stroke nurse is important in the early management of stroke patients and has some referential value to promote in the country. Although our study has demonstrated the benefits and feasibility to some point, more clinical samples and studies are needed to confirm in the future.

## Data Availability

The datasets used and/or analyzed during the current study are available from the corresponding author on reasonable request.
